# Channel-Blind Joint Source–Channel Coding for Wireless Image Transmission

**DOI:** 10.3390/s24124005

**Published:** 2024-06-20

**Authors:** Hongjie Yuan, Weizhang Xu, Yuhuan Wang, Xingxing Wang

**Affiliations:** State Key Laboratory of Media Convergence & Communication, Communication University of China, Beijing 100024, China

**Keywords:** wireless image transmission, joint source–channel coding, attention mechanism, broadcasting

## Abstract

Joint source–channel coding (JSCC) based on deep learning has shown significant advancements in image transmission tasks. However, previous channel-adaptive JSCC methods often rely on the signal-to-noise ratio (SNR) of the current channel for encoding, which overlooks the neural network’s self-adaptive capability across varying SNRs. This paper investigates the self-adaptive capability of deep learning-based JSCC models to dynamically changing channels and introduces a novel method named Channel-Blind JSCC (CBJSCC). CBJSCC leverages the intrinsic learning capability of neural networks to self-adapt to dynamic channels and diverse SNRs without relying on external SNR information. This approach is advantageous, as it is not affected by channel estimation errors and can be applied to one-to-many wireless communication scenarios. To enhance the performance of JSCC tasks, the CBJSCC model employs a specially designed encoder–decoder. Experimental results show that CBJSCC outperforms existing channel-adaptive JSCC methods that depend on SNR estimation and feedback, both in additive white Gaussian noise environments and under slow Rayleigh fading channel conditions. Through a comprehensive analysis of the model’s performance, we further validate the robustness and adaptability of this strategy across different application scenarios, with the experimental results providing strong evidence to support this claim.

## 1. Introduction

**Figure 1:**
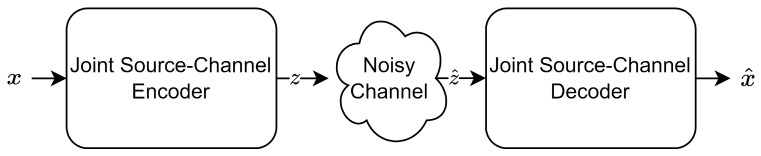
Illustration of the DL-based JSCC.

The assumption underlying Shannon’s theorem is that source and channel coding can be independently performed without compromising information transmission efficiency. However, practical constraints such as complexity and latency often make it impractical to use very large block lengths. In light of this, the design of a joint source–channel coding (JSCC) scheme has been shown to outperform separate source–channel coding in terms of performance, particularly in the finite block length regime [1,2,3,4,5,6,7]. The advent of advanced deep learning technology has sparked considerable interest among researchers in data-driven DL-based JSCC approaches [8,9,10,11,12,13,14,15,16,17,18]. The intelligent and concise design paradigm of DL-based JSCC aligns seamlessly with the fundamental principles of communication. Figure 1 illustrates the newly proposed DL-based JSCC algorithm [13]. These novel approaches use an implicit model, where explicit source and channel encoding schemes are replaced by neural networks that map pixel values to the channel input signal. This mapping process is achieved through an encoder, which converts the source data into a channel input signal, and a decoder, which reconstructs the source data from the channel output signal. The encoder and decoder are trained together to optimize the system’s overall performance.

In recent years, many JSCC methods based on deep learning (DL) techniques have been proposed. Bourtsoulatze et al. were the first to apply a DL-based JSCC model to wireless channel image data transmission in their study [13]. This study suggests that DL-based JSCC methods can effectively avoid the cliff effect compared to traditional SSCC methods. To further improve image transmission quality, Kurka et al. [14] proposed deepjscc-f, a DL-based JSCC model that incorporates a feedback mechanism, inspired by the Schalkwijk–Kailath scheme [19]. Additionally, Wang et al. [11] introduced an iterative receiver for image reconstruction within the DL-based JSCC framework, building upon the concept of turbo codes. This iterative process greatly enhances the overall performance of image reconstruction. Some studies have also improved model transmission performance by dynamically adjusting the resolution [20] and transmission rate [21].

Despite the impressive performance of these DL-based JSCC approaches, a limitation arises from the strict requirement for the model to precisely match the SNR of the current communication channel. To address this issue, Xu et al. [9] proposed the Attention DL-based JSCC (ADJSCC) approach. ADJSCC aims to enable a single model to dynamically adapt to different channel conditions. Specifically, in ADJSCC, an attention fusion (AF) module incorporates the channel SNR as prior information into both the channel encoder and decoder. This allows the network to adapt to varying SNR conditions. ADJSCC has inspired subsequent research on channel-adaptive techniques [22,23,24,25]. However, the ADJSCC approach still faces challenges in real-world scenarios. First, the ADJSCC model requires accurate SNR estimation to adapt to the wireless channel, which is difficult to achieve due to the impact of channel estimation mismatch [26]. Second, the ADJSCC encoding process relies on feedback channel information, making it suitable only for point-to-point communication scenarios. Although Ding et al. [16] proposed a channel-adaptive JSCC (CaJSCC) scheme, which makes it a feasible solution for multi-user scenarios, it comes with the drawback of significantly reduced performance. Additionally, it still relies heavily on accurate channel estimation.

We observe that previous work adheres to traditional communication theory, which posits that while a feedback link cannot increase the channel capacity of the noisy forward link, it can significantly reduce the coding effort needed to attain a specific performance level [19]. However, in DL-based JSCC, or semantic communication, the objective is no longer based on the correct delivery of symbols. Numerous studies have shown that DL-JSCC can overcome the cliff effect, maintaining the ability to transmit content even when channel capacity is severely limited. This suggests that DL-JSCC methods can adapt to different SNR levels. Yin et al. introduced a DL-based channel estimation method that operates without the need for pilot data [27], showcasing the neural network’s strong learning capability with respect to channel characteristics. However, current methods that explicitly encode SNR into the model overlook the neural network’s adaptability to noise at different SNRs in semantic transmission tasks.

In this paper, we propose a novel Channel-Blind JSCC (CBJSCC) method that can adapt to varying channel conditions without the need for channel estimation. CBJSCC adapts to varying SNRs intrinsically through the network, instead of explicitly encoding information into the model. This approach benefits from not suffering performance degradation due to SNR estimation mismatches, promising higher robustness in practical applications.

We observe that the performance of some existing DL-based JSCC models significantly benefits from structural improvements in the model. Therefore, the CBJSCC model incorporates a meticulously designed network that integrates convolution-based attention and self-attention mechanisms in both the encoder and decoder. We evaluate the performance of CBJSCC, and the results indicate that CBJSCC exhibits outstanding performance in additive white Gaussian noise (AWGN) and slow Rayleigh fading channels. Specifically, in the AWGN channel, CBJSCC outperforms existing feedback-based DL-based JSCC methods that currently exhibit the best performance. This advantage is achieved with reduced computational costs and a smaller number of parameters.

The main contributions of this study can be summarized as follows:

- We introduce CBJSCC, a channel-adaptive JSCC method that does not rely on channel state information. CBJSCC leverages the inherent learning capability of networks to adapt to wireless channels with varying SNRs, requiring less stringent communication environments. With these characteristics, CBJSCC avoids the detrimental effects of channel mismatch on model performance and is suitable for a broader range of communication scenarios, such as broadcast and deep-space communications.
- In CBJSCC, we propose encoders and decoders designed with local attention and self-attention mechanisms. This design ensures a wider receptive field for the network and enhances model performance. Under equivalent transmission quality, CBJSCC has advantages over ADJSCC in both computational and parameter efficiency.
- We analyze the impact of SNR on CBJSCC. The results show that the benefits of including SNR information are very limited for CBJSCC. This provides valuable insights for the further exploration of channel adaptability issues in semantic communications.
- Considering the alignment of the model with data from different domains in the real world, we train on the ImageNet dataset and evaluate CBJSCC on datasets from various fields. The results demonstrate that CBJSCC has robust domain adaptability, making it suitable for transmission tasks across different domains.

The subsequent sections of this paper are structured as follows: In Section 2, we provide an overview of the related research to CBJSCC. Section 3 outlines the CBJSCC model that we propose. Following that, in Section 4, we present the results of our experiments along with a comprehensive analysis. Lastly, we offer our concluding remarks in Section 5.

## 2. Related Works

### 2.1. Autoencoder

The Autoencoder (AE) [28,29] aims to discover a compressed representation of the input data while minimizing the discrepancy between the input and its reconstructed output. AEs have gained prominence in deep learning [30,31,32], proving highly effective for data compression and feature extraction, particularly in JSCC. The primary objective of AE is to learn the mappings between the input space, denoted as X, and the feature space, denoted as Z. The class of functions that map elements from X to Z is denoted as F, while the class of functions that remap elements from Z back to X is denoted as G. The distortion function, represented by Δ, can take discrete or continuous forms. The autoencoder problem can be formulated as follows:

(1) $$\begin{array}{cc} \; & F:X\to Z\\ \; & G:Z\to X\\ \; & \left(f^{*},\;g^{*}\right)=\arg\min_{f\in F,g\in G}\Delta \left(X,\;g\left[f\left(X\right)\right]\right) \end{array}$$

In this study, we specifically focus on the auto-associative case, where the input and output features are identical.

The Denoising Autoencoder (DAE) [33] is particularly relevant to JSCC, as it aims to capture stable structures, such as dependencies and regularities, inherent in the observed input distribution. The objective of DAE can be formulated as follows:

(2) $$\left(f^{*},\;g^{*}\right)=\arg\min_{f\in F,g\in G}\Delta \left(X,\;g\left[f\left(\hat{X}\right)\right]\right)$$

The key distinction between DAE and conventional AE lies in the data used for training. DAE derives its input data from a corrupted version, denoted as $\hat{x}\in \hat{X}$, obtained by randomly corrupting the authentic data *x* using a corruption process defined as $\hat{x}=x⊙q_{D}\left(\hat{x}|x\right)$. This corruption process enables the model to learn a more robust representation. In contrast, JSCC focuses on data reversibility, reconstruction quality, and the utilization of channel characteristics in compression processing. DAE, on the other hand, prioritizes the precision of reconstructing the original content over the stability or reconstructability of the signal.

### 2.2. Attention Mechanism

The attention mechanism is a fundamental concept in deep learning, allowing for the efficient scanning of global information while focusing on specific target regions. Various attention mechanisms, such as channel attention, spatial attention, or a combination of both, enable the refinement of feature maps through independent reweighting [34,35,36,37,38]. By assigning weights to channels or spatial locations on the feature maps, these mechanisms enhance the receptive field of convolutional networks, enabling a more focused analysis of significant local areas within the input data.

Given an intermediate feature map $W_{in}\in R^{C\times H\times W}$ as input, the final output vector $W_{out}$ is computed using the following equation:

(3) $$W_{out}=M\left(W_{in}\right)⨂W_{in}$$

In Equation (3), $A=M(W_{in})$ represents the attention map.

Self-attention represents a distinct version of the attention mechanism [39]. The issue with convolution-based attention is its inability to capture long-range dependencies. Conversely, self-attention provides a more flexible strategy, adept at recognizing both local and global dependencies. The computation process can be represented as:

(4) $$\begin{array}{cc} \mathrm{Attention}(QW^{Q},\;KW^{K}) & =\mathrm{softmax}\left(\frac{\left(QW^{Q}\right){\left(KW_{K}\right)}^{T}}{\sqrt{d}}\right)\\ W_{out} & =\mathrm{Attention}\left(QW_{Q},\;KW_{K}\right)\left(VW_{V}\right) \end{array}$$

In the above equation, *Q*, *K*, and *V* are the query, key, and value vector sequences, respectively, obtained through a linear transformation of the input feature map *W*. The tensor dimension is denoted by *d*. The softmax function is applied to the dot product of the query and key vectors, and the resulting attention weights are utilized to compute a weighted sum of the value vectors.

Multi-Head Self-Attention (MSA) is a more intricate form of the attention mechanism. It incorporates multiple attention heads in self-attention models to capture diverse points of interest, which is illustrated as follows:

(5) $$\mathrm{Multi}\;-\;\mathrm{Head}\left(Q,\;K,\;V\right)=\mathrm{Concat}\left({\mathrm{head}}^{1},\;{\mathrm{head}}^{2},\;\ldots ,\;{\mathrm{head}}^{h}\right)W_{O}$$

In Equation (5), $W_{i}^{Q}$, $W_{i}^{K}$, and $W_{i}^{V}$ represent the projection matrices for the *i*-th attention head out of *h* heads:

(6) $${\mathrm{head}}^{i}=\mathrm{Attention}\left(QW_{Q}^{i},\;KW_{K}^{i}\right)VW_{V}^{i}$$

The integration of MSA can improve the model’s expressive capacity by allowing it to gather different information through separate attention heads, extracting more comprehensive and nuanced features.

The incorporation of the self-attention mechanism in computer vision tasks has shown promising results, although it comes with certain drawbacks. Notably, the self-attention mechanism can increase both training and inference time, requiring substantial computational resources [40,41,42]. However, recent research has made remarkable strides by integrating the self-attention mechanism with convolutional networks. This integration aims to address these challenges by reducing computational complexity and introducing additional inductive biases [43,44,45]. By calculating dot-product attention scores and performing weighted summation, the self-attention mechanism generates a more concise sequence representation. This representation effectively minimizes redundancy while preserving the original features’ semantic information, making it more suitable for storage, processing, and transmission purposes.

## 3. Proposed Method

### 3.1. Overall Architecture

**Figure 2:**
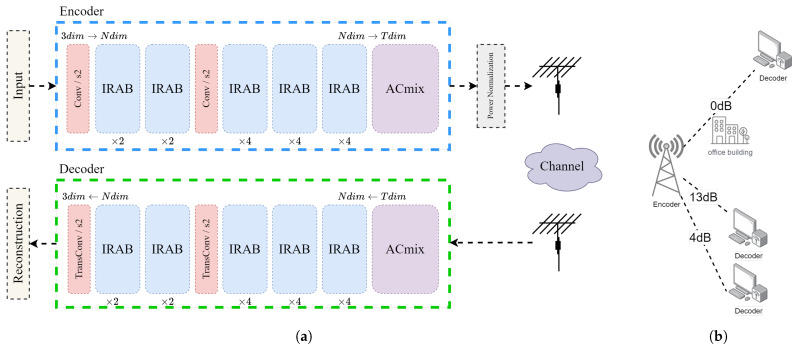
(**a**) Architecture of the proposed CBJSCC method. The blue dashed box and the green dashed box represent the encoder and the decoder, respectively. The numbers under each IRAB block indicate the expansion factor. *N* and *T* represent the dimension of the feature map. (**b**) In real-world deployment of the CBJSCC model, the encoded signal can be transmitted directly to the client through various channels at different ratios.

This section presents a comprehensive description of the CBJSCC method as illustrated in Figure 2a. CBJSCC follows the fundamental concept of an autoencoder (AE). In this approach, we consider a wireless system for image transmission, where the image *x* has dimensions of *h* (height) × w (width) × c (channels). Here, *x* is a set of real numbers $R^{n}$, where *n* equals the product of *w*, *h*, and *c*. The encoding process, guided by parameters θ, deterministically maps the input *x* to complex symbols *z* through the function $f_{\theta }$, satisfying the following relationship:

(7) $$z=f_{\theta }\left(x\right)\in C^{k}$$

During the encoding phase, the output feature dimension of the image is mapped to a value *N*, which is set to 192 by default in this study. At the output, the channel dimension of the feature map is converted to *T*.

The encoder first employs an efficient local attention module, followed by the construction of global features using the ACMix network [46]. The encoded image is transformed into a complex channel signal $z\in C^{k}$ via a conjugate transpose, where *k* represents the dimension of the encoded complex symbols. Here, *n* is termed the source bandwidth, *k* is termed the channel bandwidth, and the ratio k/n is referred to as the bandwidth compression ratio (CBR).

The transmission rate in the CBJSCC method is adjusted by altering the dimension *T* of the final self-attention module. Since the encoder includes two downsampling operations with a stride of 2, the formula for calculating *k* is $T\;\times \;n/\left(2\;\times \;16\;\times \;c\right)$. With *c* generally set to 3, the bandwidth compression of CBJSCC corresponds to T/96. The model can be trained under AWGN or fading channel conditions, including Rayleigh fading channels, using suitably simulated channel conditions. The received signal is modeled as:

(8) $$\hat{z}=hz+\omega$$

Here, *h* denotes the channel fading coefficient (or channel gain), sampled from an independent and identically distributed complex Gaussian distribution. $\omega \in C^{k}$ represents circularly symmetric complex Gaussian distribution noise. For an AWGN channel, *h* is equal to 1. The received signal $\hat{z}$ is then sent to the decoder, which maps it back.

The encoder and decoder of the CBJSCC are symmetrically designed, with the decoder serving as the mirror image of the encoder, and parameterized by $\theta^{\prime }$:

(9) $$\hat{x}=g_{\theta^{\prime }}\left(\hat{z}\right)\in R^{n}$$

CBJSCC is explicitly designed for image transmission over dynamic wireless channels, circumventing the need for Channel State Information (CSI) estimation. Consequently, *h* and ω remain unknown to both the encoder and decoder. This process can be formulated as:

(10) $$\begin{array}{cc} \theta^{★},\;\theta^{\prime ★} & =\underset{\theta ,\theta^{\prime }}{\arg\min}\frac{1}{n}\sum_{i=1}^{n}L\left(x^{\left(i\right)},\;{\hat{x}}^{\left(i\right)}\right)\\ \; & =\underset{\theta ,\theta^{\prime }}{\arg\min}\frac{1}{n}\sum_{i=1}^{n}L\left(x^{\left(i\right)},\;g_{\theta^{\prime }}\left(h*f_{\theta }\left(x^{\left(i\right)}\right)+\omega \right)\right) \end{array}$$

Here, *L* denotes the loss function, while $\theta^{★}$ and $\theta^{\prime ★}$ represent the optimal parameters of the encoder and decoder, respectively.

When the value of *h* is set to 1 and ω is set to 0, CBJSCC aligns with the architecture of a traditional AE.

Prior works such as DeepJSCC and ADJSCC were designed for point-to-point communication scenarios. For example, DeepJSCC requires switching to a corresponding model based on the channel’s SNR, while ADJSCC can only encode and decode for a single channel SNR. Serious performance degradation occurs when the estimated SNR does not match the actual SNR. In broadcast and other one-to-many communication scenarios, the encoded signal needs to be transmitted over multiple wireless channels with different SNRs. CBJSCC, however, does not require encoding and decoding for specific SNRs; instead, it can adapt to various SNRs, making it more suitable for broadcast communication than other methods. This design makes CBJSCC particularly well suited for broadcast communication as shown in Figure 2b.

### 3.2. The Inverted Residual Attention Bottleneck

**Figure 3:**
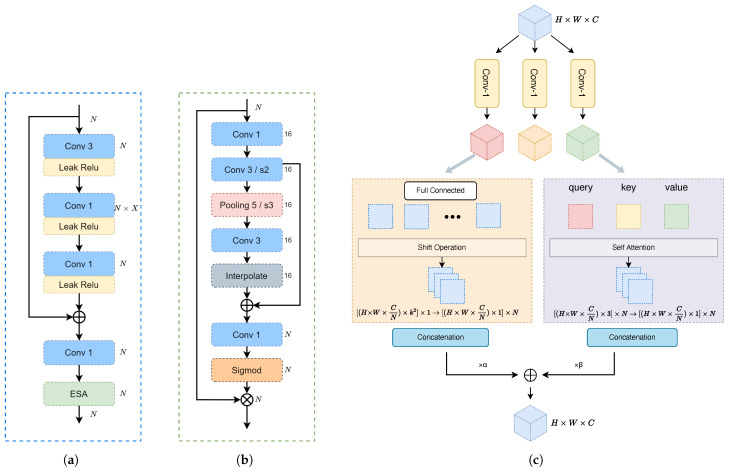
(**a**) The IRAB module is characterized by the convolution kernel size, denoted by the numbers in the box. In IRAB, the variable *X* signifies the multiple of the current convolution kernel size in relation to the input. (**b**) The ESA block. (**c**) The ACMix block.

Recent research indicates that ConvNext can deliver enhanced performance in smaller-scale networks [47]. Inspired by the concept of the inverted bottleneck residual structure in ConvNext and some image super-resolution tasks [48], we introduce the Inverted Residual Attention Bottleneck (IRAB) structure as illustrated in Figure 3a. Here, the input features first pass through a convolution layer with a kernel size of 3 × 3. Next, the dimension of the convolution layer is expanded by *X* times through an extended 1 × 1 convolution layer. Finally, a convolution layer is used to restore the tensor to its original dimensions. Excluding the bias term, the computational complexity of the IRAB can be represented as the Flops ratio (2 × X + 9)/27 relative to the standard 3 × 3 residual block. In the design of the IRAB, we set the expansion factor *X* to 2 in the first downsampling module to balance the expansion of feature maps and computational efficiency. In the second downsampling module, we increase the value of *X* to 4 to enhance the network’s representational capacity at a higher spatial resolution. This choice is supported by theoretical analysis and experimental observations.

Subsequently, the feature is passed through a 1 × 1 conventional layer, before being fed into the enhanced spatial attention (ESA) modules as described in [49,50]. As depicted in Figure 3b, to decrease the computational complexity, the ESA block starts with a 1 × 1 convolutional layer that reduces the channel dimension from *N* to 16. This is followed by a convolution layer with a stride of 2 and max-pooling. This downsampling of features expands the receptive field of the convolution layers, enhancing the allocation of attention weights. To reinstate the spatial and channel dimensions of the input features, we employ interpolation and a 1 × 1 convolution layer. Subsequently, the attention weights of the input features are derived by applying the sigmoid function *M* as depicted in Equation (3).

### 3.3. Convolution and Self-Attention Mixed Module

The encoding and decoding process can suffer from compromised accuracy and effectiveness when the correlations between encoded symbols over extended distances are overlooked or diminished. We adapt a modified ACMix block [46], which combines self-attention and convolution operations to effectively capture long-range dependencies. This combination allows ACMix to handle images more effectively, capturing both the overall outline and shape of the image and fully utilizing local preferential features within the image. Another advantage is ACMix’s ability to handle input images of different sizes.

Here, we replace the batch normalization layer with generalized divisive normalization (GDN) [51]. GDN better adapts to the statistical characteristics of local filter responses, resulting in natural image blocks and improved performance.

## 4. Experiments and Analysis

### 4.1. Training and Implementation Details

We used the ImageNet [52] for training. The training process utilized a single Nvidia RTX 4090 with a batch size of 112. We randomly cropped the images into patches of size 128 × 128 during each iteration. The training SNR was uniformly sampled from −5 dB to 20 dB. The initial learning rate was set to 0.0001 and decayed exponentially based on the loss. Training stopped once the model converged. We evaluated image restoration quality using two metrics: peak signal-to-noise ratio (PSNR) and Structural Similarity Index Measure (SSIM).

We used L1 Charbonnier loss for training [53]. L1 Charbonnier promotes robustness to noise and outliers compared to the standard L1 loss function [54,55]. Let *x* and $\hat{x}$ denote the original image and the reconstructed image, respectively. The L1 Charbonnier loss function is defined as follows:

(11) $$L\left(\hat{x},\;x\right)=\sqrt{{\left(x-\hat{x}\right)}^{2}+\epsilon }$$

Here, ϵ is a small value to ensure the Charbonnier penalty is never zero.

Our experiment uses the recently proposed efficient optimization algorithm, EvoLved Sign Momentum (Lion) [56]. Our research indicates that the Lion optimizer can speed up the model’s convergence.

### 4.2. Experiment and Results

**Figure 4:**
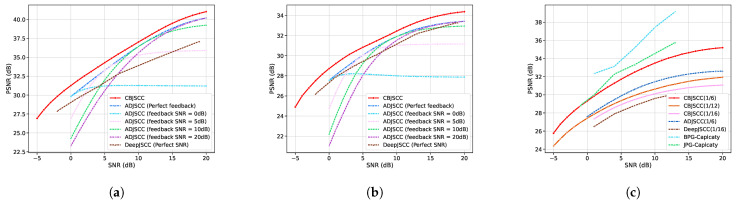
Image reconstruction performance comparison on the Kodak dataset under an AWGN channel. (**a**,**b**) display results for transmission rate ratios of 1/6 and 1/12, respectively. The solid red curve represents the performance of the proposed CBJSCC method. The dashed lines represent the performance of ADJSCC under different SNR feedback conditions, including perfect feedback and feedback SNRs of 0 dB, 5 dB, 10 dB, and 20 dB. (**c**) extends the evaluation to slow Rayleigh fading channels, comparing CBJSCC with alternative methods, indicated within parentheses, at different transmission rates.

The results in Figure 4 highlight the superior performance of the CBJSCC method across various scenarios. We did not include DeepJSCC in the comparison, as ADJSCC has already surpassed DeepJSCC. We also did not compare with other recent models, as those works focus on other functionalities, not channel adaptivity.

Figure 4 presents a comparative analysis of image reconstruction performance using the Kodak dataset under an AWGN channel and slow Rayleigh fading channels. The panels of Figure 4a,b illustrate the results for transmission rate ratios of 1/6 and 1/12, respectively, highlighting the robustness of the proposed CBJSCC method. The solid red curve denotes the superior PSNR values of CBJSCC across various SNR levels, outclassing ADJSCC and DeepJSCC, the latter of which was omitted from this comparison due to the previously established dominance of ADJSCC.

However, real-world communication often accompanies issues of SNR estimation mismatch. The dashed lines in the panels of Figure 4a,b depict the performance of ADJSCC under varying feedback SNR conditions, ranging from ideal (perfect feedback) to a practical scenario with estimation errors (feedback SNR = 0 dB, 5 dB, 10 dB, and 20 dB mismatches). These lines reveal a pronounced degradation in the image reconstruction quality of ADJSCC as the feedback SNR estimation error increases, particularly at lower SNR levels. The design of CBJSCC negates the need for explicit SNR encoding, leveraging the neural network’s inherent adaptability to channel variations. This characteristic provides a significant advantage in real-world communications, where SNR estimation mismatch is a common challenge, thereby enhancing the robustness of CBJSCC compared to ADJSCC.

Figure 4c extends the evaluation to slow Rayleigh fading channels. Here, CBJSCC demonstrates exceptional adaptability by modifying the transmission rate, achieving the highest PSNR values across all examined SNR levels compared to other DL-based methods. Notably, CBJSCC with a ratio of 1/6 leads the performance. The comparison also includes the performance of traditional image compression algorithms—JPG and BPG—encoded under 16QAM modulation at a transmission rate of 1/6. The performance of CBJSCC in fading channels is on par with or superior to JPG-Capacity at low SNR values. However, its effectiveness decreases marginally when the SNR exceeds 1 dB. BPG, known for its high compression efficiency, is used as a reference point, but a significant performance decline in actual environments restricts its direct comparison. In summary, the empirical results corroborate the enhanced adaptability and robustness of CBJSCC in diverse communication environments, establishing its potential for real-world applications where channel conditions are dynamic and unpredictable.

**Figure 5:**
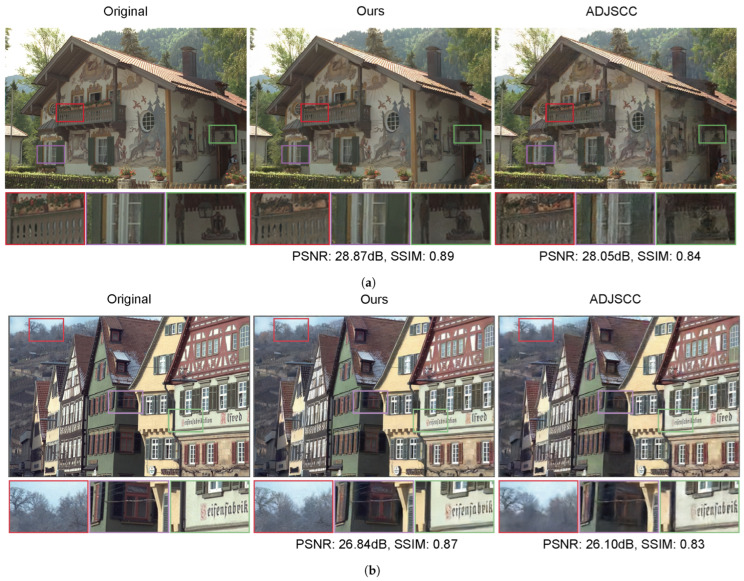

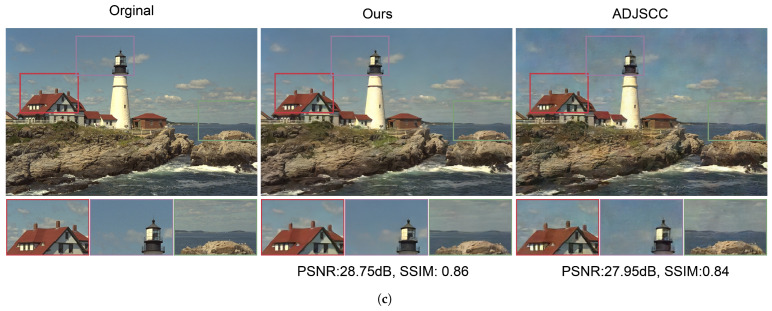
(**a**,**b**) demonstrate the image reconstruction performance of CBJSCC and ADJSCC on the Kodak dataset under an AWGN channel with a 1 dB SNR at different transmission rates: (**a**) 1/6 and (**b**) 1/12. (**c**) shows the image reconstruction under a slow Rayleigh fading channel with a transmission rate of 1/6.

Figure 5 provides a visual comparison of the two methods. Here, we only consider the case where the ADJSCC model uses perfect SNR feedback. Both CBJSCC and ADJSCC demonstrate impressive overall reconstruction capabilities. By observing the magnified box in Figure 5a, it is evident that CBJSCC can accurately recover the details and structure of the original image, while ADJSCC introduces blurring and distortion. In Figure 5b, the distant branches in the highlighted red box of the image reconstructed by ADJSCC appear blurry, losing many details. In contrast, CBJSCC preserves the clear texture of these branches, making the image more realistic. From these observations, we can infer that CBJSCC provides superior image reconstruction quality compared to ADJSCC, retaining more details in texture-rich areas.

### 4.3. Exploring Model Capacity

The previous discussion highlighted the CBJSCC model’s strong feature extraction and reconstruction capabilities, which are mainly due to its design. In this section, we will further examine the connection between expressive capacity and effective model complexity within the CBJSCC model. Expressive capacity refers to a model’s ability to approximate complex problems, while effective model complexity measures the usable capacity of a trained model [57]. Expressive capacity is the maximum knowledge a model architecture can encompass. Investigating model complexity is important to understand the depth of a deep model and explore fundamental problems related to JSCC tasks. The model’s capacity can be adjusted by changing the value of *N* in the IRAB block as shown in Figure 2, while keeping the transmission dimension *T* constant.

We assessed the performance of the CBJSCC model across various *N* values while maintaining a constant transmission rate of 1/6, comparing it to ADJSCC. Table 1 presents our evaluation results, with the best PSNR values highlighted in red and the second-best in blue.

As the *N* value increases, the CBJSCC model’s performance improves significantly. For example, the PSNR at SNR 1 dB increases from 28.53 dB for CBJSCC(64) to 31.90 dB for CBJSCC(192). This trend shows that higher model capacity allows for better reconstruction quality. When compared with ADJSCC, CBJSCC(128) achieves a PSNR of 34.71 dB at SNR 7 dB with 3.21 million parameters and 182.05 G MACs. In contrast, ADJSCC achieves a PSNR of 34.59 dB with 10.76 million parameters and 398.18 G MACs. CBJSCC(128) not only reduces parameters by 70.16% and computational effort by 54.28% but also achieves better or comparable PSNR across all SNR levels. WITT has 28.20 million parameters and 99.00 G MACs, much higher than CBJSCC models. Despite its high parameter count, the WITT image reconstruction quality is comparable to that of CBJSCC(128) but lower than that of CBJSCC(192) and CBJSCC(256) at high SNR levels. CBJSCC(192) and CBJSCC(256) offer superior performance and resource efficiency, particularly at higher SNR levels, showing better scalability and adaptability.

Our results show that increasing model capacity enhances performance without a proportional increase in computation and storage requirements. The adaptability of CBJSCC to channel variations without considering SNR underscores its potential for scalable applications in varying communication environments. CBJSCC models, particularly with higher *N* values, demonstrate that superior performance can be achieved with fewer resources, making them ideal for practical applications, where computational and storage efficiency are critical. Overall, the CBJSCC models exhibit a significant advantage in both performance and efficiency, affirming their potential for practical deployment in JSCC tasks.

### 4.4. Impact of Channel SNR Information

In previous experiments, we demonstrated that our proposed CBJSCC model performs better in an additive white Gaussian noise (AWGN) channel, even without SNR information. This suggests that neural networks can adapt to channel characteristics. To further analyze the impact of SNR information on the model, we compared several different schemes.

**Figure 6:**
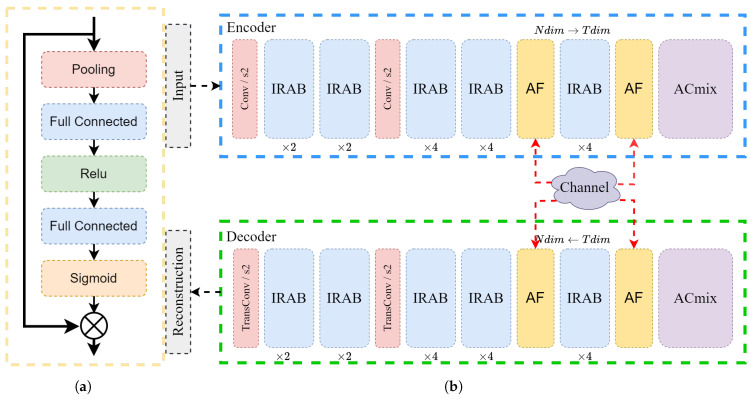
(**a**) Architecture of the AF module in ADJSCC. (**b**) Modified CBJSCC with the AF module. The red dashed line represents the input of SNR information. The yellow block represents the inserted AF module.

We introduced the Attention Feature (AF) module from ADJSCC, incorporating channel information into the encoder and decoder components as shown in Figure 6a. The AF module acts as a channel attention mechanism, integrating channel SNR information into the network. Figure 6b shows how we inserted the AF module into the framework. We discuss three scenarios next. In the first scenario, both the encoder and decoder can obtain the current SNR. In the second scenario, only the decoder can obtain the current SNR, with no channel feedback. In the third scenario, neither the encoder nor the decoder can obtain the current channel SNR, which is the situation in this study.

**Figure 7:**
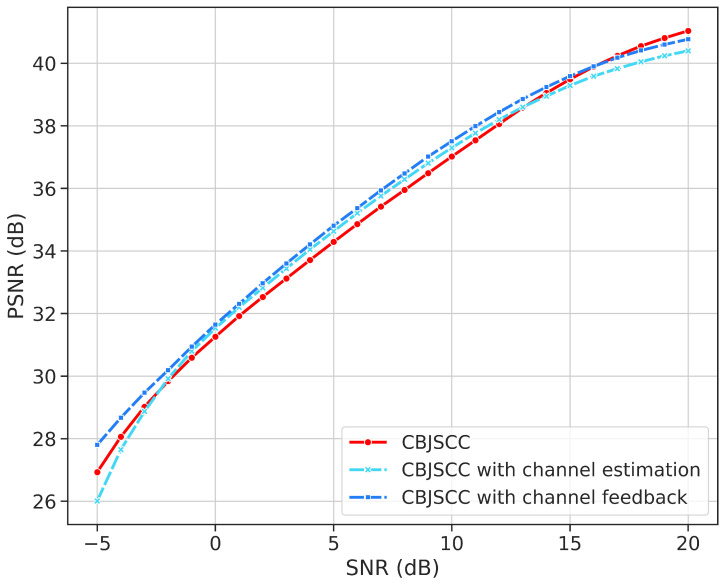
PSNR curves for reconstructed images under three different scenarios.

According to the results in Figure 7, different model approaches show some variation in performance at low SNR with channel feedback. However, through repeated experimentation, we found that this variation may be due to the inherent unpredictability of deep learning models. Overall, in the AWGN channel, no method was significantly superior to others. Therefore, our research findings indicate that introducing SNR estimation and feedback does not significantly improve the performance of deep learning-based JSCC.

This study also suggests that learning-based JSCC methods can implicitly learn and adapt to various channels. They can automatically adjust transmission rates based on changes in channel quality. It is important to note that, as deep learning models are black boxes, we cannot directly observe their internal mechanisms. These findings may provide insights for exploring new joint encoding and decoding methods and emphasize that the design of channel feedback mechanisms needs to align with specific scenarios.

### 4.5. Adaptation Ability of Different SNR Ranges

The previous results indicate that this model can adapt to different SNR levels and outperforms ADJSCC in terms of performance. Now, we will explore the impact of adaptability to the SNR range. In previous experiments, we trained the model with SNR uniformly distributed between −5 dB and 20 dB. For comparison, we introduced three additional training strategies. The first experiment involved training individual models, each using one SNR value from the set {1, 4, 7, 10, 13}. The second experiment used discrete SNR values randomly selected from the set {1, 4, 7, 10, 13} during training. Finally, the third experiment used a continuous uniform sampling technique within the range [1, 13] to determine the training SNR values.

**Figure 8:**
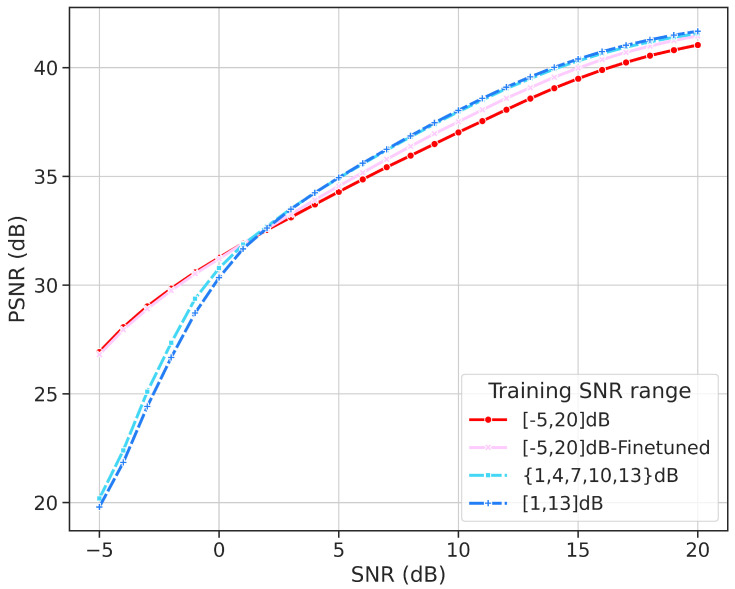
Performance comparison with different training SNR ranges. The red solid line represents the model trained on a wide SNR range from [−5, 20] dB. The pink dashed line indicates the same range but with fine-tuning. The light blue dotted line shows the model trained on discrete SNR values {1, 4, 7, 10, 13} dB. The blue dash–dot line represents the model trained on the continuous range [1, 13] dB.

Figure 8 illustrates that the performance curves for both discrete and continuous SNR values largely overlap, indicating no substantial differences within the examined range. However, restricting the training SNR to [1, 13] dB improves the model’s performance compared to the broader [−5, 20] dB range, due to better reconstruction within a smaller, more consistent SNR range. This advantage is offset by a decrease in image reconstruction quality for SNR values below 1 dB. This is understandable, as the model can more easily reconstruct the original image from signals with SNR changing within a smaller range. Of course, this advantage comes at the cost of sacrificing image reconstruction quality below 1 dB SNR.

In Table 2, we compare models trained with multiple different SNR values separately with models trained with SNR randomly sampled within these ranges. As expected, the model can achieve the best image reconstruction quality when the training and testings SNRs are equal. When the SNR exceeds 10 dB, we found that the second-best model is not the one trained using the discrete random sampling strategy. This may suggest that JSCC models can benefit from training at high SNR. We fine-tuned the model trained within [1, 13] dB under [−5, 20] dB, resulting in the fine-tuned model as shown in Figure 8. The performance of the model fine-tuned based on [1, 13] dB surpassed the model directly trained in the [−5, 20] dB range within the 1 to 13 dB range.

### 4.6. Domain Adaptation Ability

Our objective is to create a model that can generalize effectively across diverse domains. However, deep learning often requires domain adaptation or transfer, which entails training a model on one domain’s dataset and evaluating it on a different domain’s dataset. In this study, we assess the CBJSCC model’s performance across different data domains, a critical factor in determining its robustness and adaptability.

**Figure 9:**
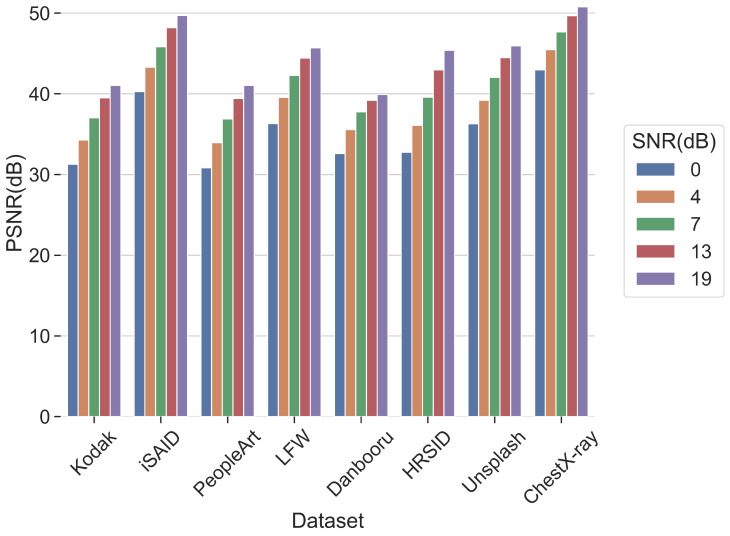
A clustered column chart showing the performance of CBJSCC across multiple dataset domains. The color of the line indicates the channel SNR.

The CBJSCC model was initially trained using the ImageNet dataset and subsequently tested on a selection of diverse test datasets as illustrated in Figure 9. These datasets cover a wide range of scenarios. The iSAID dataset [55] consists of high-resolution aerial images used for instance segmentation. The People-Art dataset [54] is employed for object detection and contains 43 different styles of people, significantly differing from standard photographs. The Labeled Faces in the Wild (LFW) dataset [52] comprises over 13,000 face images downloaded from the internet, exhibiting substantial variations in pose, lighting, and facial expression, making it a prominent dataset for face recognition in computer vision. The Danbooru2021 dataset [53] is a vast collection of anime-style images, encompassing 4.9 million illustrations. The High-Resolution SAR Images Dataset (HRSID) [59] is utilized for ship detection and segmentation in high-resolution Synthetic Aperture Radar (SAR) images, which include SAR images with different resolutions, polarizations, and ports. The Unsplash Dataset [60] is an extensive collection of high-quality images from professional photographers worldwide, covering a broad range of camera brands, models, lenses, and focal lengths. The Chest X-ray8 dataset [61] is a medical dataset comprising a large number of X-ray images.

The results show that CBJSCC consistently performs well across different domains. Remarkably, CT images, being an exceedingly rare type, witnessed the model attaining a reconstruction performance of 50 dB PSNR on the CT Medical dataset. This could be attributed to the smoother color and texture variations in CT images and their fewer high-frequency details. Similarly, the CBJSCC model performed exceptionally well on the iSAID dataset, which contains a large number of water areas with minor frequency changes. The Danbooru2021 and People-Art datasets, which encompass art and cartoon images and typically exhibit strong artistic styles and visual effects, such as bright colors, unique compositions, and smooth lines, showed slightly lower reconstruction performance from the CBJSCC model. Nevertheless, the model still achieved a reconstruction performance of over 30 dB PSNR at an SNR level of 0 dB on these datasets.

The experimental results demonstrate that CBJSCC maintains excellent reconstruction capability across diverse datasets, including some rare data types. This suggests that similar DL-based JSCC methods possess robustness in wireless image transmission tasks.

## 5. Conclusions

In this study, we introduce the Channel-Blind Joint Source–Channel Coding (CBJSCC) method, a novel technique for wireless image transmission that leverages the adaptive capabilities of deep learning to accommodate various channel conditions without relying on explicit SNR knowledge.

The CBJSCC technique employs a carefully designed encoder–decoder structure enhanced with attention mechanisms, outperforming existing deep learning-based joint source–channel coding strategies without the need for channel feedback. The effectiveness of CBJSCC highlights the potential of deep learning-based JSCC methods to autonomously identify and adapt to the intrinsic characteristics of communication channels, thereby mitigating the adverse effects of channel noise and associated errors. Experimental results have confirmed the scalability and resilience of the CBJSCC method across multiple domain datasets, emphasizing its versatility and wide applicability. The contributions of this paper are multifaceted: (1) the introduction of an innovative deep learning-based JSCC technique that does not depend on specific SNR information, (2) demonstration of the technique’s superiority in image transmission performance, and (3) validation of its generalizability across various communication scenarios.

Future research directions include integrating CBJSCC with advanced signal processing techniques to enhance its performance in challenging channel scenarios, investigating the adaptability of CBJSCC to dynamic channel environments, which requires continuous learning and channel model updates, and expanding the applicability of CBJSCC to other media types, including video and audio, thereby broadening its use in various communication fields such as broadcasting, deep space communication, and undersea communication. We expect this research not only to enrich the JSCC field but also to inspire future efforts to overcome the limitations of traditional communication methods, thereby promoting the development of more efficient and reliable wireless multimedia transmission systems.

## Figures and Tables

**Table 1:** EMC analysis across different model capacity.

Model(N dim)	Params(M)	MACs(G)	PSNR(dB)				
			SNR 1 dB	SNR 4 dB	SNR 7 dB	SNR 13 dB	SNR 19 dB
CBJSCC(64)	0.87	48.10	28.53	29.36	29.90	30.04	30.54
CBJSCC(96)	1.85	104.26	30.55	32.39	34.06	36.55	37.72
CBJSCC(128)	3.21	182.05	30.95	32.88	34.71	37.85	39.72
CBJSCC(160)	4.96	281.49	31.30	33.14	34.90	37.96	39.76
CBJSCC(192)	7.09	402.57	**31.90**	**33.71**	35.41	38.58	40.81
CBJSCC(256)	12.49	709.66	31.81	33.66	**35.45**	**38.70**	**40.97**
ADJSCC [9]	10.76	398.18	30.53	32.60	34.59	38.00	40.02
WITT [58]	28.20	99.00	31.01	32.29	34.36	36.54	37.62

**Table 2:** Reconstructed image PSNR with different training SNR ranges.

Channel SNR	Training SNR Range					
	{1, 4, 7, 10, 13} dB	1 dB	4 dB	7 dB	10 dB	13 dB
1 dB	31.86	**32.08**	30.31	26.29	23.77	22.27
4 dB	34.24	33.37	**34.28**	33.27	30.88	28.70
7 dB	36.20	33.85	35.61	**36.46**	36.07	34.66
10 dB	37.97	34.09	36.25	37.92	**38.53**	38.22
13 dB	39.49	34.20	36.57	38.73	39.99	**40.33**

## Data Availability

The original contributions presented in the study are included in the article, further inquiries can be directed to the corresponding author.
